# A Shift in Paradigms: Spatial Genomics Approaches to Reveal Single-Cell Principles of Genome Organization

**DOI:** 10.3389/fgene.2021.780822

**Published:** 2021-11-19

**Authors:** Andres M. Cardozo Gizzi

**Affiliations:** Centro de Investigación en Medicina Traslacional Severo Amuchastegui (CIMETSA), Instituto Universitario de Ciencias Biomédicas de Córdoba (IUCBC), CONICET, Córdoba, Argentina

**Keywords:** chromatin 3D architecture, chromosome conformation, topologically associated domain (TAD), fluorescence *in situ* cell hybridization (FISH), oligopaint, transcriptional regulation, genome organization, stochasticity

## Abstract

The genome tridimensional (3D) organization and its role towards the regulation of key cell processes such as transcription is currently a main question in biology. Interphase chromosomes are spatially segregated into “territories,” epigenetically-defined large domains of chromatin that interact to form “compartments” with common transcriptional status, and insulator-flanked domains called “topologically associating domains” (TADs). Moreover, chromatin organizes around nuclear structures such as lamina, speckles, or the nucleolus to acquire a higher-order genome organization. Due to recent technological advances, the different hierarchies are being solved. Particularly, advances in microscopy technologies are shedding light on the genome structure at multiple levels. Intriguingly, more and more reports point to high variability and stochasticity at the single-cell level. However, the functional consequences of such variability in genome conformation are still unsolved. Here, I will discuss the implication of the cell-to-cell heterogeneity at the different scales in the context of newly developed imaging approaches, particularly multiplexed Fluorescence in situ hybridization methods that enabled “chromatin tracing.” Extensions of these methods are now combining spatial information of dozens to thousands of genomic *loci* with the localization of nuclear features such as the nucleolus, nuclear speckles, or even histone modifications, creating the fast-moving field of “spatial genomics.” As our view of genome organization shifts the focus from ensemble to single-cell, new insights to fundamental questions begin to emerge.

## Introduction

In eukaryotes, DNA is arranged in a three-dimensional (3D) packaging within the nucleus. The genome hierarchical 3D organization conforms a key regulatory layer of gene expression and cell fate control (Bonev and Cavalli, 2016). Individual chromosomes are spatially partitioned into discrete “chromosome territories” (Cremer and Cremer, 2001; Bolzer et al., 2005; Cremer et al., 2006). Down from the chromosomal scale, the genome is partitioned into two types of structural units. On the one hand, active (A) and inactive (B) compartments are genomic regions spanning several mega-base pairs (Mb) which tend to engage in homotypic (A-A or B-B) rather than heterotypic contacts. On the other hand, topologically associating domains (TADs) are defined as regions at the sub-Mb scale displaying higher intra-domain interactions and relatively insulated from neighboring domains.

The segregation of active and repressed chromatin was observed for the first time by Emil Heitz, who in 1928 suggested the terms “heterochromatin” and “euchromatin” (Passarge, 1979). A great deal about chromatin spatial organization has been learned thanks to the development of biochemical methods called chromatin conformation capture (3C) and 3C derivatives (Goel and Hansen, 2021; Jerkovic and Cavalli, 2021). 3C-based techniques rely on DNA crosslinking to fix the interacting sequences and nuclease fragmentation to retrieve the contact frequency of pairs of genomic positions. In particular, genome-wide maps of chromatin interaction have been obtained by sequencing-based high-throughput chromosome conformation capture techniques (Hi-C). Through initial Hi-C maps, it was found that domains sharing biochemical properties such as epigenetic marks and transcriptional status tend to interact with domains of the same type, to form A/B compartments (size ∼ 1–3 Mb), which resemble euchromatin and heterochromatin, respectively (Lieberman-Aiden et al., 2009).

The other genome “structural unit,” TADs, were discovered due to an increased genomic resolution of 3C-based methods (Dixon et al., 2012; Nora et al., 2012; Sexton et al., 2012), with an average size between 185–900 kb in mammals (Dixon et al., 2012; Rao et al., 2014; Bonev et al., 2017) and 100–150 kb in *Drosophila* (Ulianov et al., 2016; Wang et al., 2018). TADs organization is, for the most part, stable between cell types or through differentiation (i.e., most TAD borders are invariant) (Dixon et al., 2015; Dixon et al., 2016). Furthermore, TADs borders coincide to a high degree with replication domain boundaries (Pope et al., 2014; Dixon et al., 2016; Ulianov et al., 2016). Even more importantly, *cis-*regulatory elements that direct transcription are mostly restricted to interactions within a TAD (Lupiáñez et al., 2015; Dixon et al., 2016). All in all, this points to a role of TADs as conserved genome “units of regulation” or even thought as physical globular domains present in most cells of a population. As we will see from single-cell techniques, the latter is an oversimplification.

Finally, the spatial compartmentalization of nuclear events is evidenced by the spatially defined localization of processes. The existence of diverse nuclear bodies, membraneless compartments with specific tasks, is a key aspect of the nuclear organization (Misteli, 2005; Mao et al., 2011). For example, nuclear speckles are subnuclear bodies that contain mRNA processing and splicing factors (Galganski et al., 2017). It has been shown that highly transcribed Pol II regions organize around nuclear speckles, whereas inactive genomic regions are frequently associated with the nuclear periphery (Guelen et al., 2008) or the nucleolus (Quinodoz et al., 2018). Inter-chromosomal contacts are organized around nuclear bodies to create a higher-order genome organization. Additionally, another principle of non-random nuclear architecture is the radial organization model where euchromatic regions (A compartment) organize centrally with respect to nuclear lamina whereas heterochromatin (B compartment) is associated with the nuclear periphery and perinucleolar regions (Buchwalter et al., 2019; Crosetto and Bienko, 2020). More importantly, the non-random organization of the genome has meaningful effects on its function and activity. As technology develops, both imaging- and sequencing-based, there is a notorious shift in paradigm: ensemble measurements are just simply not enough to understand the structure-function relationship. Here I will discuss the microscopy improvements that lead to new insights into the stochasticity in genome organization and its influence on the mechanisms involved.

## Introducing “Spatial Genomics”

Microscopy methods enable the visualization of genomic features in single cells (Xie and Liu, 2021). Fluorescence *in situ* hybridization (FISH) detects the physical position of targeted sequences by the annealing of labeled DNA or RNA probes. As genome-wide methods started to be widely used across many laboratories, single-cell 3D-DNA FISH was used as an orthogonal method to validate observations (Nora et al., 2012). Therefore, selected pairs of *loci* were used to measure physical distances and compare them with 3C contact frequencies (Giorgetti and Heard, 2016).

Two major FISH limitations can be identified when it comes to extending its throughput. The first is the probe design and production. Traditionally, FISH probes are derived from molecular cloning to vectors such as bacterial artificial chromosomes (BACs) (Roohi et al., 2008) and PCR-based methods like HD-FISH (Bienko et al., 2013). These methods are laborious and time-consuming, especially to produce multiple probes. Due to advances in high-throughput parallel chemical synthesis, it is now possible to construct FISH probes from oligonucleotides (oligos), termed Oligopaints (Beliveau et al., 2012; Beliveau et al., 2015). Oligo-based probes are selected bioinformatically and allow for great flexibility in terms of experimental design, targeting from a few kilobases (kb) to Mbs (Beliveau et al., 2018).

The other limitation is the color channels available to imaging, restricting FISH to 2–3 *loci* per experiment. An initial effort using a sequential color code trace a whole chromosomal arm (Lowenstein et al., 2004) although it has remained challenging to unambiguously identify multiple *loci*. Xiaowei Zhuang’s lab developed the concept of sequential imaging of target *loci* combining the flexibility of Oligopaints with microfluidics in a regular widefield fluorescence microscope to accomplish the multiplexed detection of FISH probes (Wang et al., 2016).

**FIGURE 1:**
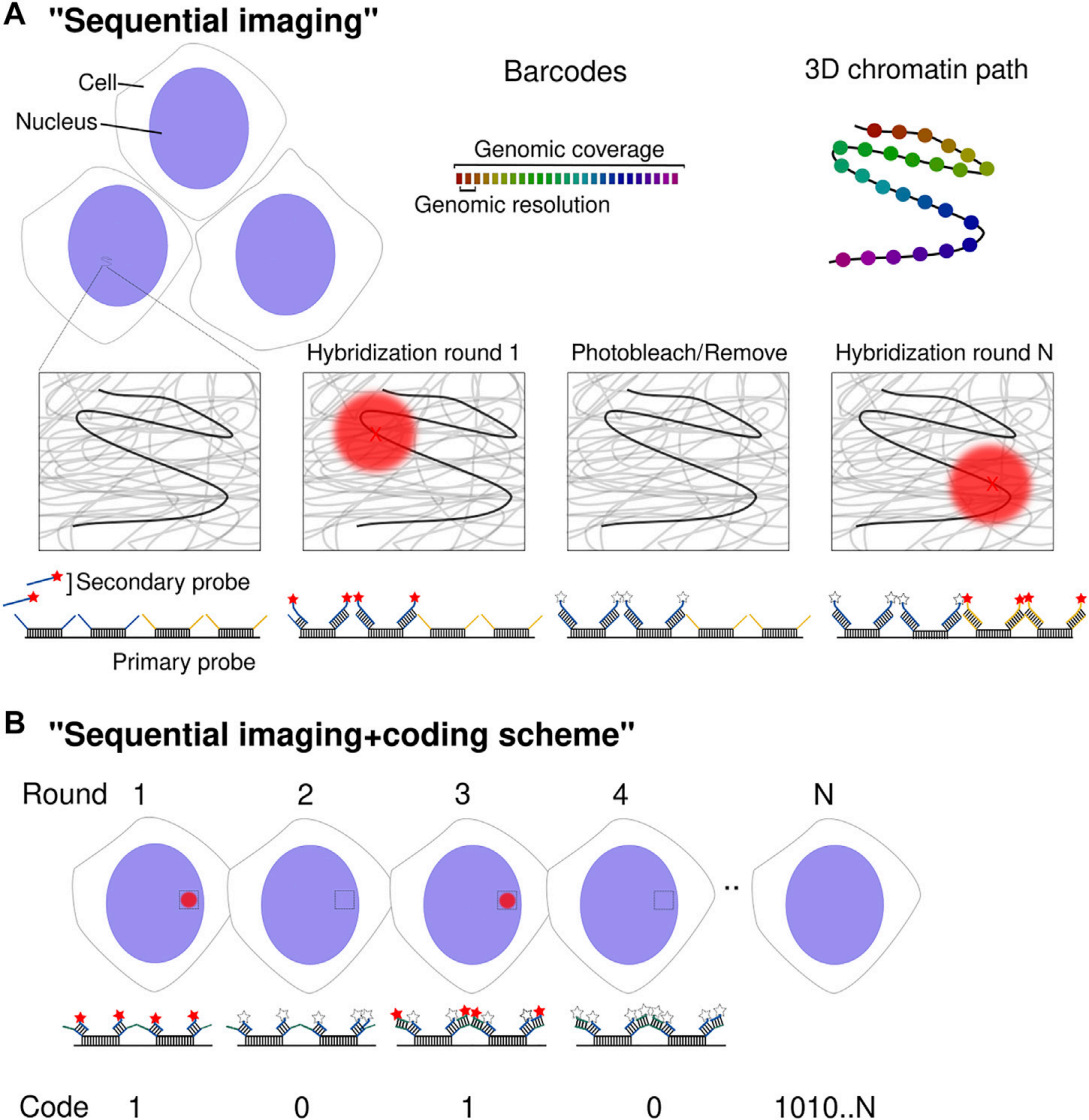
Spatial genomics approaches. **(A)** Schematic diagram of multiplexed DNA barcode detection. DNA loci are detected sequentially through secondary readout probes complementary to barcode-specific overhang sequences. The centroid of diffraction-limited spots (red X) is determined with nanometric precision. After each hybridization and imaging round, fluorophores are either removed or photobleached before starting a new cycle. Upon completion of N rounds, the chromatin path is determined in individual chromosomes across thousands of cells. **(B)** Schematic diagram of the implementation of a coding scheme using N sequential cycles. Although the procedure to determine the chromatin path is the same as in **(A)**, a coding scheme is implemented. Every barcode is detected by more than one readout probe (two in this case) by the use of multiple overhang sequences per barcode. This leads to the detection of the same barcode in several imaging rounds. Detection is read as a “1” whereas no detection as “0”. Post-signal processing allows decoding the position of 2^N^ barcodes.

The idea is to use a set of oligos (hereafter “barcode”), targeting a specific locus, that shares the same overhang region that is then recognized by a fluorescently labeled secondary oligo. After hybridizing primary probes to all target regions, barcode-specific secondary probes are injected to then imaged across multiple fields of view, photobleach and start a new hybridization cycle (Figure 1A). In each cycle, the barcodes appear as fluorescent spots whose centroid position is determined with nanometric precision (Boettiger and Murphy, 2020). Therefore, the method enables a direct tracing of the chromatin path with a genomic coverage and resolution according to the design of the Oligopaint probes (i.e., size of the barcoded regions and the distance between barcodes).

The initial approach managed to image ∼30 genomic *loci* covering a whole human chromosome with a genomic resolution around the Mb and revealing that at this scale there is a strong correlation between mean spatial distance with Hi-C contact frequency (Wang et al., 2016). Following this study, three papers appeared within a 6-months window, further developing the multiplexed methods using “chromatin tracing” (Bintu et al., 2018), “Hi-M” (Cardozo Gizzi et al., 2019) and “optical reconstruction of chromatin architecture” (ORCA) (Mateo et al., 2019) and achieved a resolution of 2–30 kb at the sub-TAD scale to cover ∼20–70 regions. At this scale, it was found that TADs, discovered by 3C-based methods, indeed appeared when averaging the population chromatin spatial conformation (see below *Stochasticity in Genome Organization*). Furthermore, it was then possible to establish, in the same cells, the transcriptional status by imaging RNA species (Cardozo Gizzi et al., 2019; Mateo et al., 2019). In *Drosophila*, it was shown that active transcription is associated with the unfolding of the gene-containing TAD at the ensemble level (Cardozo Gizzi et al., 2019). In eukaryotic cells, transcription is controlled by *cis*-regulatory elements (CREs) such as enhancers, silencers and promoters. By using contiguous barcodes to achieve a resolution of ∼2 kb, it was possible to study CRE hubs that regulate gene expression. It was found that enhancer-promoter (E-P) distance was only a weak predictor of transcription (Mateo et al., 2019) and that distal CRE hubs are formed before gene activation (or even TADs) and may reinforce transcriptional repression (Espinola et al., 2021). Additionally, the simultaneous detection of RNA can also be used as a proxy to perform cell-type specific studies (Mateo et al., 2019; Liu et al., 2020; Espinola et al., 2021).

The “coding scheme” concept was later introduced to deliver throughput orders of magnitude higher. It was adapted from multiplexed error-robust FISH (MERFISH) (Chen et al., 2015) or sequential FISH (seqFISH) (Lubeck et al., 2014; Shah et al., 2018; Eng et al., 2019), initially developed for RNA *in situ* detection. Xiaowei Zhuang’s DNA-MERFISH and Long Cai’s seqFISH+ were developed in parallel and consist of embedding a particular barcode with more than one class of readout sequence, constituting a binary code. In other words, two to five different overhang sequences are added in each barcode, that will be then detected with multiple readout fluorescent oligos (Figure 1B). The “1” or “0” value of each bit corresponds to the presence or not of a particular barcode in a hybridization round. This allows for 2^N^ genomic positions to be imaged in N rounds of hybridization. The vast majority of possible encoded barcodes are not used to implement an error detection and correction scheme. Su et al. (2020) employed 100-binary barcodes with two “1” bits and 98 “0” bits to image 1,041 genomic *loci* employing 50 hybridization rounds in two channels. In this study, a particular genomic locus would be decoded after being detected (*on* or “1”) in a particular spatial localization in two out of 100 different hybridization cycles. In Takei et al. (2021a) 2,460 genomic loci were imaged using 80 hybridization rounds in two channels.

A different spatial genomics approach is the combination of microscopy and sequencing by adapting and improving fluorescence RNA *in situ* sequencing (IGS) or FISSEQ technology (Lee et al., 2014). Recent developments of IGS have permitted both targeted (Oligo-FISSEQ) (Nguyen et al., 2020) or untargeted approaches (Payne et al., 2021). Oligo-FISSEQ uses barcoded Oligopaints targeting multiple genomic regions that are sequenced *in situ* whereas untargeted IGS uses Tn5 transposase to randomly incorporate DNA sequencing adaptors into fixed DNA, achieving a resolution of ∼1 Mb genome-wide. Finally, combining chromating tracing with multimodal RNA- and immuno-labeling (Liu et al., 2020; Su et al., 2020; Payne et al., 2021; Takei et al., 2021a) enables the profiling of genome conformation, nuclear bodies, gene expression and epigenetic status in the same cell.

In the last 3 years, this revolution kickstarted a new field. These very recent developments put us within range of genome-wide spatial maps of chromatin organization, complementing the best of genomics and microscopy fields. More and more labs are developing and implementing “spatial genomics” approaches even if at the present the methodology employs custom-made setups and requires an in-house knowledge of automated image analysis. From these approaches, the different contributions of heterogeneity to chromosome architecture at different scales are being sorted out.

## Stochasticity in Genome Organization

Genome organization has a large degree of variability at the single-cell level (Finn and Misteli, 2019) and the 3D segregation of chromosomes shows a clear variability between cells. Accordingly, the relative position of a particular chromosome to each other is not “predefined” yet the “chromosome territories” are physical structures present in all cells within a population. This is not the case for A/B compartments or TADs that arise from averaging multiple cell conformations in mammalian cells. In other words, they are statistical features of genome organization not necessarily present from cell to cell. Here I will discuss the evidence supporting this claim, mainly obtained from spatial genomics techniques unless stated otherwise.

The segregation of active and inactive chromatin by the preferred contacts between chromatin of the same class is observed in single cells, that display their chromosomes in a “polarized fashion” in interphase human fibroblasts (Wang et al., 2016; Nir et al., 2018) and *C. elegans* embryos (Sawh et al., 2020). This indicates that compartments, or regions of active/inactive chromatin, are localized side-by-side with various degrees of intermixing. Consistent with genome-wide studies, chromatin tracing experiments found a spatial correlation between nucleoli and nuclear lamina with B-compartment regions (Liu et al., 2020; Su et al., 2020) or between speckle with A-compartment regions (Liu et al., 2020). Furthermore, the degree of segregation between compartments showed a gradual establishment during the cell cycle, increasing from G1 to G2/S phase (Su et al., 2020) as previously seen by Hi-C (Abramo et al., 2019). However, individual chromosomes display a high level of variation, from the extreme complete segregation of A- and B-clusters to a highly intermingling configuration (Liu et al., 2020; Su et al., 2020).

**FIGURE 2:**
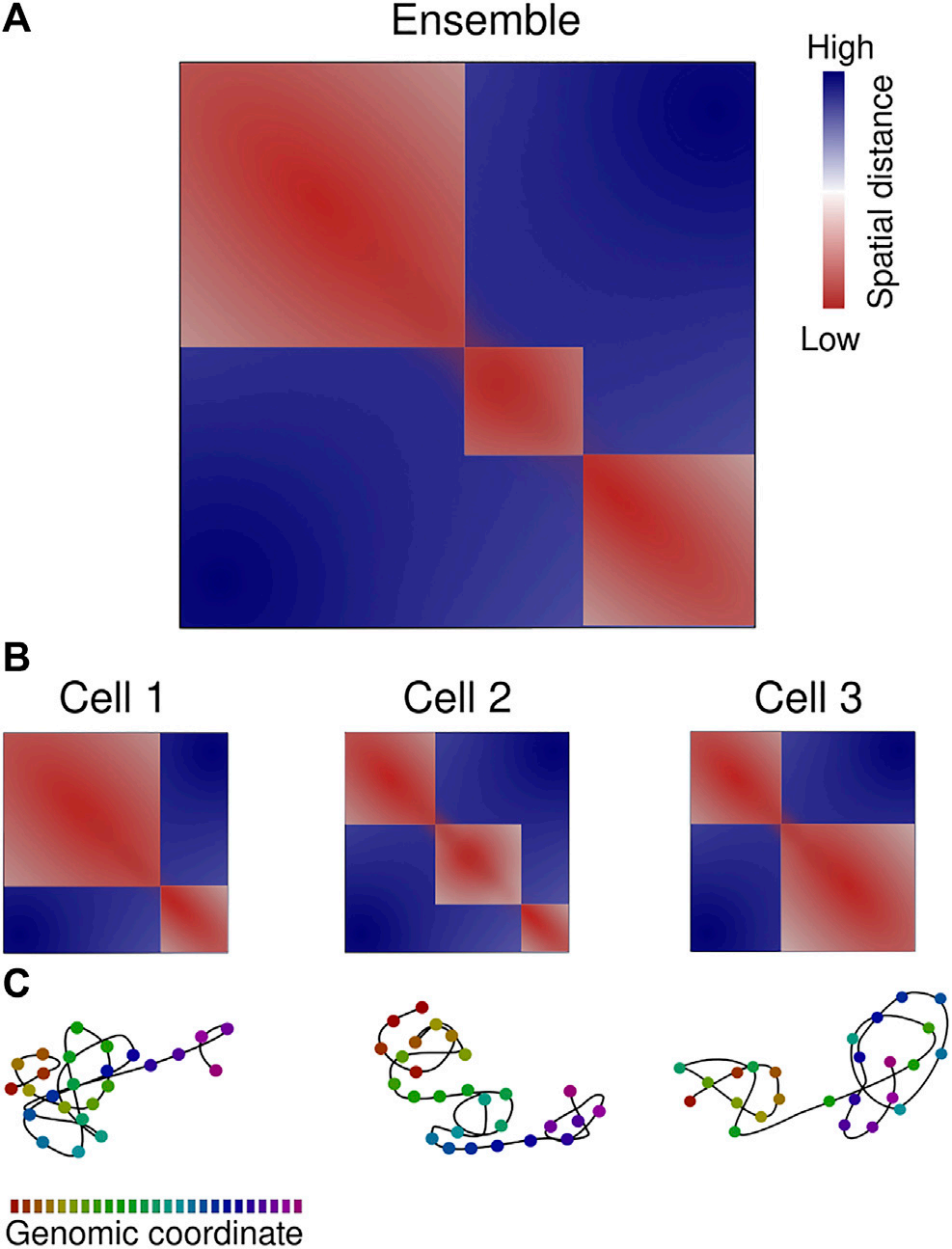
Chromatin organization is variable between cells. **(A)** Ensemble-averaged median spatial distance map, color-code from red to blue according to the scale bar. Three eTADs are clearly visible. **(B)** Single-cell spatial distance maps indicate the heterogeneity in chromatin 3D architecture. **(C)** Chromatin path representation of the single-cell distance maps, color-coded according to the genomic coordinate scale bar. Representation based on spatial genomic approaches.

Microscopy reports determined a low contact probability (1–10%) of long-range associations between any pair of loci and a modest two-fold increase within TADs (Cattoni et al., 2017; Finn et al., 2019). The single-cell contact maps frequently exhibit TAD-like structures, as seen in multiple chromatin tracing studies (Bintu et al., 2018; Mateo et al., 2019; Su et al., 2020; Payne et al., 2021; Takei et al., 2021b). These are local physical domains of enhanced contact that are well separated from one another. The physical properties of domains, such as size or degree of insulation, displayed a large heterogeneity (Boettiger et al., 2016; Nir et al., 2018; Szabo et al., 2018; Luppino et al., 2020; Szabo et al., 2020). This is consistent with the high variability in TAD formation observed in single-cell sequencing-based biochemical methods (reviewed in Ulianov et al., 2017; Galitsyna and Gelfand, 2021), such as Hi-C (Flyamer et al., 2017; Nagano et al., 2017; Stevens et al., 2017; Tan et al., 2018), ChIA-Drop (Zheng et al., 2019) or scSPRITE (Arrastia et al., 2021). Consistently, the boundaries of such domains do not necessarily correspond to ensemble-averaged TADs (eTADs; Bohrer and Larson, 2021) (Figure 2).

In mammals, Hi-C-defined eTADs are frequently flanked by pairs of CTCF binding sites in convergent orientation and serve as anchors for chromatin loops (Nora et al., 2012; Rao et al., 2014). TAD-like domain boundaries were preferentially positioned at CTCF and cohesin binding sites, belonging to eTADs boundaries, peaking at ∼15% probability. However, all other genomic loci within a TAD shared a boundary probability of ∼5–7% (Bintu et al., 2018; Su et al., 2020). In contrast, *Drosophila* TADs, whose borders are not enriched in CTCF, are much more stable from cell-to-cell, observed both by microscopy (Szabo et al., 2018) and single-cell Hi-C (Ulianov et al., 2021); although the reasons are under investigation (Ulianov and Razin, 2021).

A very graphical example of TAD architecture at the single-cell level is this is the organization of the inactivated X chromosome, used as a model for chromosome organization (Galupa and Heard, 2018). In mammalian females, the two copies of X chromosomes display a very different transcriptional and epigenetic landscape. At the ensemble level, the inactivated X chromosome (Xi) displays only two mega domains with the boundary located at macrosatellite DXZ4. Strikingly, both the active X chromosome and Xi show TAD-like domains at the single-cell level (Cheng et al., 2021; Takei et al., 2021b).

The role of TADs in transcription regulation is still an open question, but evidence supports both a role on facilitating CREs communication within the TAD and on blocking enhancer-promoter contacts between TADs (Furlong and Levine, 2018; Cavalheiro et al., 2021). However, the stochastic nature of TADs (and compartments) questions the real influence of TADs on transcription modulation (Bohrer and Larson, 2021). The timescales involved in chromosome organization and transcription is a dimension that needs to be considered, and that is not being addressed by FISH or sequencing-based methods in fixed cells (Nollmann et al., 2021). The live-cell tracking of loci gives information on the dynamic nature of regulatory DNA contacts such as E-P interactions (Brandão et al., 2021), and thus can bring understanding into the role of 3D genome organization in CREs regulation (Schoenfelder and Fraser, 2019).

Cell-to-cell variability within a phenotypically indistinguishable population has also been found in the transcriptome and epigenome (DNA methylation and histone modification profile) (Golov et al., 2016). For example, results by microscopy indicate that mRNA levels of targeted genes fluctuate from cell to cell due to the intrinsically stochastic, infrequent events of gene activation (Raj et al., 2006). Transcriptional activation can reposition genes in space (Zink et al., 2004), possibly by the action of RNA polymerases (Heinz et al., 2018; Brandão et al., 2019). Moreover, chromatin marks exhibit high variability between cells (Takei et al., 2021a), such as the intensities of H3K4me3 histone mark at different gene bodies (Woodworth et al., 2021), that at some point may regulate chromatin compartmentalization (Wang et al., 2019) (see below). Moreover, H3K4me3 histone mark intensities at different gene bodies show great heterogeneity (Woodworth et al., 2021) or that chromatin marks exhibit high variability in embryonic stem cells (Takei et al., 2021a). Considering that transcriptional activation can reposition genes in space (Zink et al., 2004) by the action of RNA polymerases (Heinz et al., 2018; Brandão et al., 2019) or that histone modifications may regulate chromatin compartmentalization (Wang et al., 2019) (see below). Therefore, the variability in gene expression and/or epigenetic status could have a direct effect on the observed stochasticity in genome conformation at the compartment and TAD levels (Ulianov and Razin, 2021). The influence and interdependence between genome organization, epigenomics and transcription is a central question in cellular biology. In the next section, I will address this by dissecting the current knowledge on the cellular processes directing them.

## Molecular Mechanisms of Spatial Organization

The two types of 3D organization found in mammalian chromosomes form by independent mechanisms (Schwarzer et al., 2017; Nuebler et al., 2018). In contrast to what was once thought, there is no hierarchy between compartments and TADs, but rather a competition between two different organization modes. The self-organization principle of the genome (Misteli, 2020) indicates that chromatin of the same type tends to interact in the space and this is directly related to the polymeric nature of the genome, although the mechanism remains elusive. Polymer physics has modeled the genome as consecutive blocks of alternating active/inactive chromatin (block copolymers), that assemble to generate the observed compartmentalization (Jost et al., 2014; Hildebrand and Dekker, 2020). It has been proposed that such compartments can arise through polymer phase separation mediated by associations of chromatin domains of similar epigenetic and/or transcriptional state (Rowley et al., 2017; Cook and Marenduzzo, 2018; Erdel and Rippe, 2018). Furthermore, a recent Hi-C study of outstanding sequencing deep revealed that median size of A/B compartments intervals is only 12.5 kb, and that even kilobase-sized domains show enhanced interactions with regions of the same class (Gu et al., 2021). However, the molecular bases of these associations are unknown.

Although spatial genetics approaches established that interactions between compartments vary from cell to cell, B-B domain contact frequencies were higher than A-A domains at distances below 75 Mb but not at higher genomic distances (Su et al., 2020), consistent with Hi-C studies in mammalian (Abramo et al., 2019) or *Drosophila* cells (Ulianov et al., 2021). The latter indicates that the mechanism of compartment segregation differs according to chromatin type. Accordingly, different players have been proposed, such as HP1alpha-mediated heterochromatin phase segregation (Larson et al., 2017; Strom et al., 2017) or clustering of active transcription sites (Hilbert et al., 2021). Based on polymer simulations, it was proposed that interactions between heterochromatin regions control compartmentalization over euchromatin contacts or the interaction of heterochromatin with the nuclear lamina (Falk et al., 2019). Recently, the role of homotypic repetitive elements and their RNA products has also been suggested as a mechanism of chromatin organization (Lu et al., 2021).

One spatial genomics study was able to establish a “chromatin profile” based on the multiplexed detection of several histone marks at specific DNA locus that matched ChIP-seq (Shen et al., 2012) or SPRITE measurements (Quinodoz et al., 2018) at 1-Mb resolution, but in this case with single-cell information. This analysis found “fixed” loci that, despite the variability in genome organization, are consistently associated with particular hallmarks (e.g., nuclear speckles, H3K9me3 mark, etc.) in most of the cells (Takei et al., 2021b). The existence of such “anchoring” points on each chromosome restricts their possible conformations. Because the spatial organization of nuclear bodies is cell-type dependent, they postulate that the nuclear architecture arises from the interaction between fixed or dominant loci with them. Moreover, related cell types have similar A/B compartment organization but very different nucleolar and lamina associations (Liu et al., 2020).

The loop-extrusion mechanism (Alipour and Marko, 2012) is to date the most accepted model of TAD formation in mammalian genomes (Nuebler et al., 2018). It postulates that the ring-shaped cohesin complex acts as a molecular motor actively extruding DNA and forming increasingly long chromatin loops that are stalled at convergent CTCF sites (Sanborn et al., 2015; Fudenberg et al., 2016; Fudenberg et al., 2017). Once bound to chromatin, the cohesin ring stochastically detaches from it, giving rise to highly dynamic structures (Hansen et al., 2017). This paradigm explains the Hi-C data showing the existence of chromatin loops between eTAD boundaries that present CTCF and cohesin complexes (Rao et al., 2014; Bonev et al., 2017). Moreover, when cohesin-loading factor Nipbl is removed from mouse cells, eTAD organization is lost (Schwarzer et al., 2017). However, chromatin tracing indicates that in single cells pairs of eTADs boundaries do not show a smaller physical distance distribution compared to control loci (Su et al., 2020) but rather there is a progressive looping anchored at the stronger CTCF binding site that progresses to more and more downstream loci (Beckwith et al., 2021). More strikingly, TAD-like domains persist upon cohesin depletion, although the boundary positions are randomized (Bintu et al., 2018). In line with this, even genomic regions that do now display eTADs form domain-like structures indicating that the folding of chromatin into this architecture is an intrinsic characteristic and that loop extrusion is a regulator of this process.

The process of compartmentalization and TAD formation shapes the genome architecture and changes the chromatin accessibility of genes and regulatory elements, modulating the functional output of genomes (Rowley and Corces, 2018). Among different cell types, the general principles of single-cell genome organization delineated above are conserved. However, cell-type specific spatial configurations delineate the functional differences (Liu et al., 2020; Takei et al., 2021b). Based on microscopy observations, we have proposed through the concept of “modulated stochasticity” that subtle changes in interaction frequencies give rise to measurable differences in genome architecture and could have a meaningful role in gene regulation (Cattoni et al., 2017). Complementary, nuclear structures such as speckles, which in practice act as chromatin scaffolds, might define different cell types and states. The stochasticity of genome architecture is a consequence of its polymeric nature, and it is modulated by several mechanisms mediated by proteins that interact through the sequence information. These mechanisms include, but are not restricted to, the processes of compartmentalization and loop extrusion. In general, sequences encode information for specific protein binding whose abundance and action will generate/regulate contacts between genomic loci.

## Conclusion and Future Perspectives

In this review, I have summarized the technological improvements and recent discoveries of spatial genomic approaches. These advances, together with single-cell sequencing methods, are shifting the focus from ensemble measurements to the organization of genomes at the single-cell level to account for the observed high degree of stochasticity and heterogeneity.

Genome organization is shaped from its polymeric nature together with biological processes such as loop-extrusion, which are both stochastic in nature. The question that emerges is what is the biological relevance of such variable organization. In other words, how the genome architecture shapes transcription: the structure/function *conundrum*. Maybe the important point here is it not anymore whether genome conformation is cause or consequence of genome function but rather what is the relationship between them. Furthermore, epigenetics and gene expression display a high degree of cell-to-cell variability. In order to reveal the contribution of each aspect to the function of genomes, new technologies capable of simultaneous detection of transcriptional output, epigenetic state and 3D conformation *in the same cell* will have to emerge. Undoubtedly, live-cell measurements, currently limited in scope, will also be necessary to understand the temporal aspects of genome organization. More importantly, despite the efforts, the function and activity of TADs and nuclear compartments continue to be unresolved. How are the specific genomic interactions generated if such heterogeneity is present? Moreover, how stochasticity is modulated to allow for precise spatio-temporal regulation of gene expression? Further developments in microscopy, genome-wide approaches and polymer simulations hold promise for the understanding of these key questions.
